# Carbon-ion radiotherapy for octogenarians with locally advanced non-small-cell lung cancer

**DOI:** 10.1007/s11604-021-01101-z

**Published:** 2021-02-19

**Authors:** Kazuhiko Hayashi, Naoyoshi Yamamoto, Mio Nakajima, Akihiro Nomoto, Hitoshi Ishikawa, Kazuhiko Ogawa, Hiroshi Tsuji

**Affiliations:** 1 QST Hospital, National Institutes for Quantum and Radiological Sciences and Technology, 4-9-1 Anagawa, Inage-ku, Chiba, 263-8555 Japan; 2 Department of Radiation Oncology, Osaka University Graduate School of Medicine, 2-2 Yamadaoka, Suita, Osaka Japan

**Keywords:** Carbon-ion radiotherapy, Locally advanced non-small-cell lung cancer, Octogenarians, Radiotherapy, Safety

## Abstract

**Purpose:**

The clinical significance of carbon-ion radiotherapy (CIRT) for octogenarians with locally advanced non-small-cell lung cancer (LA-NSCLC) remains unclear. We aimed to evaluate the clinical outcomes of CIRT alone for octogenarians with LA-NSCLC.

**Materials and methods:**

We evaluated 32 patients who underwent CIRT alone between 1997 and 2015. The median age was 82.0 years (range, 80–88 years). In terms of clinical stage (UICC 7th edition), 7 (21.9%), 10 (31.3%), 11 (34.4%), and 4 (12.5%) patients had stage IIA, IIB, IIIA, and ΙΙΙB disease, respectively. The median CIRT dose was 72.0 Gy (relative biological effectiveness), and the median follow-up period was 33.1 months.

**Results:**

All patients successfully completed CIRT. Regarding grade ≥ 2 toxicities, 1 (3.1%), 3 (9.4%), and 4 (0.7%) patients developed grade 3 radiation pneumonitis, grade 2 radiation pneumonitis, and grade 2 dermatitis, respectively. No grade ≥ 4 toxicities were observed. The 2 year LC, PFS, and OS rates were 83.5%, 46.7%, and 68.0%, respectively.

**Conclusion:**

CIRT alone is safe and effective for octogenarians with LA-NSCLC.

## Introduction

Primary lung cancer is one of the most common cancers worldwide [1]. Japan has one of the fastest aging societies, with a mean life expectancy at birth of 81 years for men and 87 years for women in 2017 [2]. Therefore, almost 40% of deaths owing to primary lung cancer are in octogenarians who are rarely eligible for highly invasive treatment modalities [3]. The standard treatment options for locally advanced non-small-cell lung cancer (LA-NSCLC) generally comprise surgery, chemotherapy, and/or radiotherapy. For inoperable patients, platinum-based chemotherapy or platinum-based chemoradiotherapy (CRT) is used. However, it sometimes induces severe hematologic toxicity, infection, esophagitis, and pneumonitis [4–6], thus making it highly toxic for octogenarians. The standard treatment modality for octogenarians with LA-NSCLC is controversial because there are limited data describing the specific therapies for patients aged 80 years or older. CRT, radiotherapy alone, and chemotherapy including an epidermal growth factor receptor tyrosine kinase inhibitor (EGFR-TKI) can be administered as treatment options [6]. In clinical practice, the treatment for octogenarians with LA-NSCLC is decided based on their performance status, comorbidity, and the patient value.

Carbon-ion radiotherapy (CIRT) is a high linear energy transfer radiotherapy that is used in some countries across Europe and Asia. CIRT has good dose-localizing properties [7], and thus can deliver a higher dose to the target volume than conventional photon radiotherapy while avoiding irradiation to the adjacent critical organs at risk, such as the lung, esophagus, trachea, and heart. CIRT alone can clinically achieve high local control (LC) rates with low toxicity [8–13]. Two published studies from our hospital have reported 2 year LC and overall survival (OS) rates of 83.5–93.1% and 51.9–68.0%, respectively, with CIRT alone in patients with LA-NSCLC [8, 13]. The incidence of grade 3–4 toxicities ranged from 3.2% to 4.9%, and none of the evaluated patients experienced grade 5 toxicities. Karube et al*.* conducted a multicenter study on CIRT alone in 64 patients with LA-NSCLC and reported similar 2 year LC and OS rates of 81.8% and 62.2%, respectively [9]. No grade ≥ 3 toxicities were observed. These clinical outcomes indicated that CIRT alone may be a safe treatment option for LA-NSCLC. However, these studies evaluated patients of all ages together, and no previous study has focused on octogenarians. Therefore, the safety and efficacy of CIRT alone for octogenarians with LA-NSCLC remain to be clarified. As such, this study aimed to investigate the clinical outcomes of CIRT alone for octogenarians with LA-NSCLC.

## Material and methods

### Study design and patients

This single-center retrospective study was approved by the Institutional Review Board of our institution (16–027) and was conducted according to the tenets of the Helsinki Declaration and its later amendments. The subjects were octogenarians who underwent CIRT alone between June 1997 and December 2015 at our institution. They were identified from a previously reported prospective phase Ι/ΙΙ study of 72 patients of all ages and a previously reported retrospective study of 69 patients of all ages who were deemed ineligible for the phase Ι/ΙΙ study at our institution [8, 13]. The studies have been described in detail previously [8, 13]. The inclusion criteria were as follows: (1) histologically or clinically diagnosed LA-NSCLC of stages ΙΙA to ΙΙΙB (the UICC’s TNM 7th Classification) [14], (2) Eastern Cooperative Oncology Group performance status of 0–2, (3) measurable tumors, (4) inoperable or refusal of surgery, (5) definitive treatments, (6) no other active cancers, and (7) no history of radiotherapy to the concerned region. The exclusion criteria included lung tumors with suspected invasion to the trachea, great vessels, heart, or carina. Consequently, data of 32 patients (18 patients from the prospective phase Ι/ΙΙ study and 12 patients from the retrospective study) who met the inclusion criteria were analyzed.

Tumor stage was evaluated using computed tomography (CT) imaging of the chest and whole abdomen, enhanced magnetic resonance imaging (MRI) of the brain, chest radiography, and blood tests. Bone scans or [18F]-fluorodeoxyglucose positron emission tomography combined with CT (18F-FDG PET/CT) were also performed. Histological or cytological diagnosis was confirmed via bronchoscopic biopsy, CT-guided biopsy, or sputum cytology in 28 (87.5%) patients and via CT, radiography, and/or 18F-FDG PET/CT in the remaining patients.

We collected information on grade ≥ 2 toxicities. Treatment-related toxicities were graded according to the National Cancer Institute’s Common Terminology Criteria for Adverse Events (version 4.0) [15].

### Carbon-ion radiotherapy

Patients were fixed using an individually tailored immobilization device (Moldcare; Alcare, Tokyo, Japan; Shellfitter; Kuraray, Osaka, Japan), and CT images were obtained in the supine or prone position using respiratory sensors to monitor the respiratory phase [7, 8]. Target delineation was performed as previously reported [8, 13]. The primary lung lesion and metastatic lymph nodes were contoured as the gross tumor volume (GTV) on CT images. The GTV with a 10 mm margin and any prophylactic lymph nodes (ipsilateral hilar and/or mediastinal lymph nodes) were defined as the clinical target volume (CTV). For N0 cases, prophylactic lymph nodes irradiation was omitted irrespective of T-stage. Planning target volume (PTV) was defined as the CTV + 5 mm safety margin. In cases where the PTV was close to the organs at risk, the margins for creating PTV was reduced.

The prescribed dose ranged from 68.0 to 76.0 Gy [relative biological effectiveness (RBE)] in 12–16 fractions, 4 days per week. Their prescribed doses were converted to 96.9 to 115.0 Gy (RBE) as the biologically effective dose using α/β = 10. A dose escalation study conducted during our investigation showed the potential of short-course CIRT alone at 72 Gy (RBE) [8], and thus the recommended dose was fixed at 72 Gy (RBE) in 16 fractions. Subsequently, this dose was adopted for all remaining patients (*n* = 21, 65.6%). The total dose was applied to the isocenter, and it enclosed the PTV conformably, with a 95% isodose line. The patients with lymph node metastasis underwent metastatic and prophylactic lymph node irradiation at a median dose of 49.5 Gy (RBE) [8, 13, 16]. The following irradiation dose constraints were applied: main bronchus, 60 Gy (RBE); esophagus, 50 Gy (RBE); and spinal cord, 30 Gy (RBE). Irradiation was performed in 2–5 fields with 250 or 290 meV carbon ions.

Regarding chemotherapy, 2 patients were administered induction chemotherapy, but none of the 32 patients received concurrent or adjuvant chemotherapy.

### Follow-up

After treatment, follow-up observations were performed at 1, 3, 6, 9, and 12 months, and every 3–6 months thereafter if serious complications had not occurred. Follow-up comprised chest CT, chest radiography, and blood tests during each evaluation. Brain MRI or F18-FDG PET/CT was performed as necessary.

### Statistical analyses

LC, progression-free survival (PFS), and OS were calculated using the Kaplan–Meier method. LC was defined as the time interval between irradiation commencement date and the local tumor regrowth at the PTV date or the last follow-up. PFS was defined as the time interval between irradiation commencement date and the date of disease progression at any site, death from any cause, or the last follow-up. OS was defined as the time interval between the start of irradiation and death or the last follow-up.

All statistical analyses were conducted using JMP statistical software (version 14.0; SAS Institute Inc., Cary, NC, USA).

## Results

### Patient characteristics

All patients successfully completed CIRT. The patient characteristics are summarized in Table 1. The median age was 82.0 years (range 80–88 years), and the median follow-up period was 33.1 months (range 2.3–150.1 months) in the overall cohort and 36.9 months for survivors. This study included 28 (87.5%) patients with performance status 1–2. There were 3 (9.4%), 12 (37.5%), 12 (37.5%), and 5 (15.6%) patients who had T1, T2, T3, and T4 disease, respectively. Furthermore, 12 (37.5%), 11 (34.4%), and 9 (28.1%) patients had N0, N1, and N2 disease, respectively. Seven patients (21.9%) had stage IIA disease; 10 (31.3%), stage IIB; 11 (34.4%), stage IIIA; and 4 (12.5%), stage ΙΙΙB. The median dose was 72.0 Gy (RBE).

**Table 1:** Patient characteristics (*n* = 32)

Factors	Value
Age (years)	
Median	82.0
Range	(80.0–88.0)
Sex	
Male	27 (84.4)
Female	5 (15.6)
PS	
0	4 (12.5)
1	26 (81.3)
2	2 (6.2)
Smoking status	
Current or previous	27 (84.4)
Never	5 (15.6)
Treatment status	
Initial treatment	30 (93.8)
Recurrence or residual cancer after chemotherapy	2 (6.2)
Location of primary tumor	
Upper lobe	24 (75.0)
Middle lobe	1 (3.1)
Lower lobe	7 (2.2)
Operability	
Yes	4 (12.5)
No	28 (87.5)
Clinical T classification	
1	3 (9.4)
2	12 (37.5)
3	12 (37.5)
4	5 (15.6)
Clinical N classification	
0	12 (37.5)
1	11 (34.4)
2	9 (28.1)
Clinical stage	
ΙΙA	7 (21.9)
ΙΙB	10 (31.3)
ΙΙΙA	11 (34.4)
ΙΙΙB	4 (12.5)
Histology of primary lung cancer	
Adenocarcinoma	15 (46.9)
Squamous cell carcinoma	11 (34.4)
Large cell carcinoma	1 (3.1)
Non-small-cell carcinoma	1 (3.1)
Unknown	4 (12.5)
Irradiated field	
Primary lesion	13 (40.6)
Primary lesion + hilar lymph nodes	4 (12.5)
Primary lesion + mediastinal lymph node	15 (46.9)
Total dose (Gy RBE)	
Median	72
Range	68–76
CTV (cm^3^)	
Median	263.8
Range	80.9–1475.5
VC (cm^3^)	
Median	2300
Range	1100–3900
FEV1.0 (cm^3^)	
Median	1300
Range	600–2700
FEV1/FVC (%)	
Median	63.8
Range	34.2–83.7
%DLCO	
Median	75.5
Range	32.8–178.4

*PS* performance status, *RBE* relative biological effectiveness, *CTV* clinical target volume, *VC* vital capacity, *FEV1* forced expiratory volume in 1 s, *FVC* forced vital capacity, *%DLCO* percent of diffusing capacity for carbon monoxide

### Toxicities

Regarding grade ≥ 2 lung toxicities, three (9.4%) and one (3.1%) patient developed grade 2 and grade 3 radiation pneumonitis, respectively (Table 2). In addition, four (0.7%) patients developed grade 2 dermatitis. No grade ≥ 2 toxicities other than dermatitis and pneumonitis were observed. Neither grade ≥ 4 nor cardiac toxicities were observed.

**Table 2:** Treatment-related toxicities

Grade	2 (%)	3 (%)	4 (%)	Total (%)
Dermatitis	4 (12.5)	0	0	4 (12.5)
Pneumonitis	3 (9.4)	1 (3.1)	0	4 (12.5)

### Local control and survival

By the end of follow-up, 8 and 17 patients had died of cancer and unrelated causes, respectively, while 7 patients were still alive. At the time of first relapse, 3 local recurrences, 5 regional recurrences (regional lymph nodes), and 4 distant metastases were detected.

**Fig. 1:**
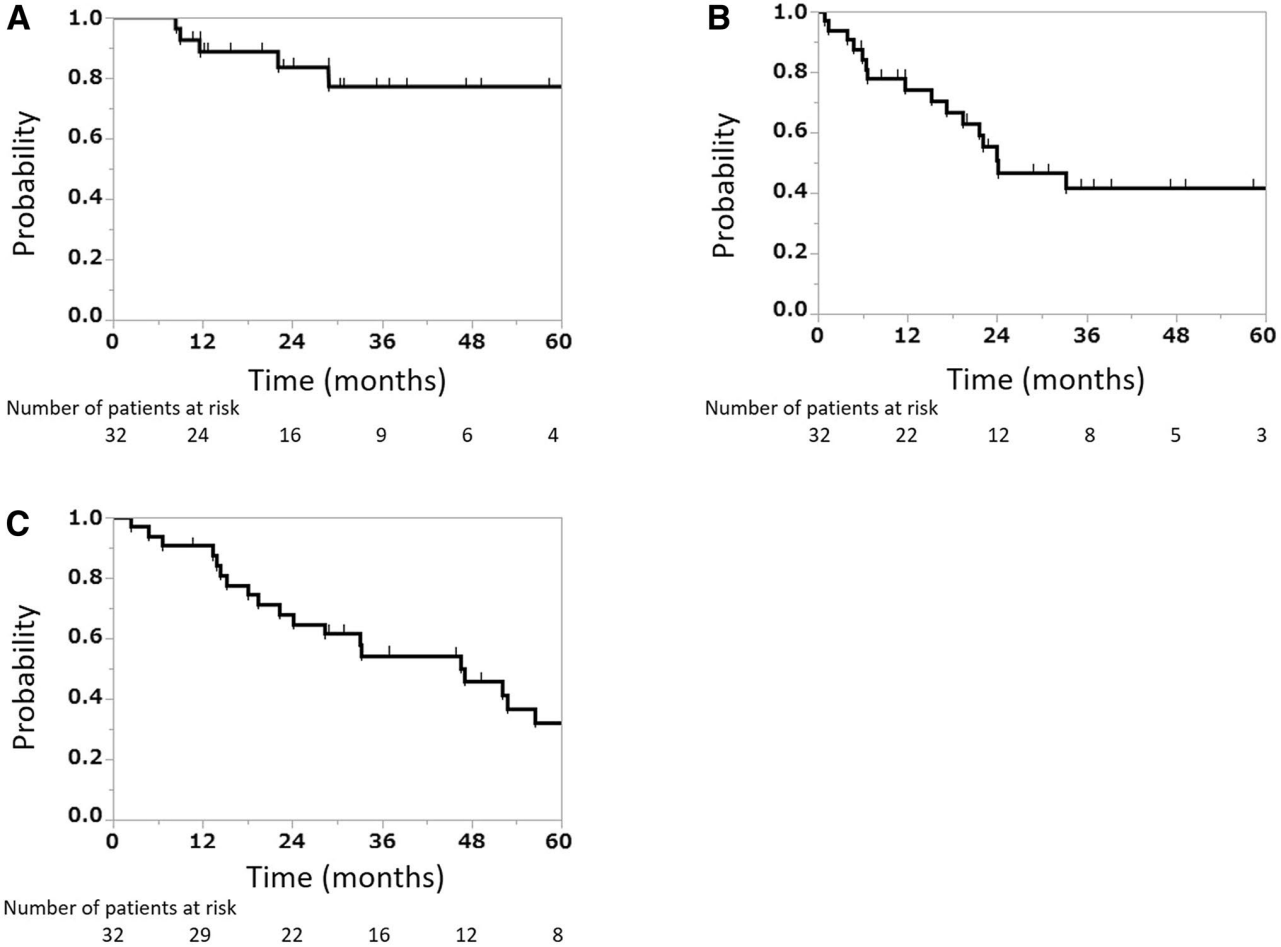
Local control rate (**a**), progression-free survival rate (**b**), and overall survival rate (**c**)

The 2 and 3 year LC rates were 83.5% [95% confidence interval (CI) 62.9–93.8%] and 77.1% (95% CI 54.3–90.5%), respectively (Fig. 1a). The 2 and 3 year PFS rates were 46.7% (95% CI 32.9–68.8%) and 41.5% (95% CI 29.2–61.2%), respectively (Fig. 1b). The 2 and 3 year OS rates were 68.0% (95% CI 50.0–81.8%) and 54.3% (95% CI 36.7–70.8%), respectively (Fig. 1c). The median PFS and OS durations were 20.8 and 33.1 months, respectively.

For survival analysis according to clinical stage, the participants were categorized into those with stages ΙΙ and ΙΙΙ LA-NSCLC, and the corresponding median PFS rates were 21.6 months and 15.1 months. Meanwhile, the corresponding median OS rates were 45.9 months and 22.2 months, respectively. In addition, the 2 year LC, PFS, and OS rates of patients with stage ΙΙ disease were 93.3%, 55.8%, and 82.4%, whereas those with stage III disease were 70.7%, 44.4%, and 50.6%, respectively.

## Discussion

CRT as a treatment option for octogenarians with LA-NSCLC induces several toxicities. However, photon radiotherapy also yields unsatisfactory outcomes, and the adoption of EGFR-TKI is rather limited. Therefore, other treatment approaches that are safer and more effective are needed for these patients. The results of this study show that CIRT alone is safe and effective for octogenarians with LA-NSCLC. To our best knowledge, this is the first study to report the clinical usefulness of CIRT alone for octogenarians with LA-NSCLC to date.

Only one study has focused on treatment options for octogenarians with LA-NSCLC [17]. In the study by Corre et al. octogenarians with EGFR-mutated stage Ι–IV lung cancer were treated using EGFR-TKI (Table 3). The main grade ≥ 3 toxicities were diarrhea (17.0% of patients), cutaneous (10.3%), and others (41.0%). With respect to photon CRT or radiotherapy alone, only studies in septuagenarians were conducted. Atagi et al*.* conducted a phase ΙΙΙ trial of photon radiotherapy with or without low-dose carboplatin for patients older than 70 years [median age 77 years (range 71–93 years)] with stage ΙΙΙ LA-NSCLC [18]. The most common grade ≥ 3 toxicity in the CRT group was leukopenia (63.5%), followed by thrombocytopenia (29.2%), infection (12.5%), radiation pneumonitis (6.5%), and dyspnea (4.2%). In contrast, the most common grade ≥ 3 toxicity in the photon radiotherapy alone group was radiation pneumonitis (5.3%), closely followed by dyspnea (5.1%) and infection (4.1%). Meanwhile, in our study, grade 3 radiation pneumonitis occurred in only 3.1% of all patients, and none of the patients developed other grade ≥ 3 toxicities. These findings indicate that CIRT alone may be safer than photon CRT or chemotherapy, including EGFR-TKI, and approximately comparable to photon radiotherapy alone with respect to grade ≥ 3 toxicity.

Several studies on photon radiotherapy alone for patients older than 70 years with stage ΙΙΙ LA-NSCLC reported that the median PFS and OS of radiotherapy alone were 6.8 months and 11–18.1 months, respectively [18–21]. Driessen et al*.* reported a median OS of 5 months when patients older than 70 years with stage ΙΙΙ LA-NSCLC selected non-curative treatment [19]. Meanwhile, although our study included only patients with N0–2 metastatic lymph node status, and consequently did not include all stage ΙΙΙ patients, the median PFS and OS for stage ΙΙΙ LA-NSCLC were 15.1 and 22.2 months, respectively, despite the median age being 82.0 years. Further, all the patients completed CIRT on schedule. These findings indicate that CIRT alone would be an effective treatment option for patients classified as N0–2.

The side effects of definitive treatment are generally more severe in elderly patients than in younger patients. Therefore, for elderly patients, the clinical goals must be decided by considering not only survival, but also quality of life and treatment tolerance. Administration of EGFR-TKI is limited to patients with NSCLC with activating EGFR mutation, although it is effective and relatively safe [22]. In addition, sustained disease control is uncommon [22]. Further, while CRT is effective, it is highly toxic for octogenarians and may worsen quality of life [21]. Unfortunately, photon radiotherapy alone also yields poor prognosis despite the relatively low incidence of severe toxicity. Meanwhile, CIRT alone achieved both a low incidence of severe toxicities and favorable local control, thus making it a promising treatment option for octogenarians with LA-NSCLC.

**Table 3:** Comparison of photon chemoradiotherapy, radiotherapy, EGFR-TKI, and CIRT alone for elderly patients with locally advanced NSCLC

Author	Median age (years)	Stage	Treatment	No. of patients	Median OS (months)	Median PFS (months)	Most common severe toxicity (%)
Atagi et al. [18]	77	ΙΙΙ	CRT	100	22.4	8.9	Grade ≥ 3 toxicity: leukopenia, 63.5%; thrombocytopenia, 29.2%; infection, 12.5%; radiation pneumonitis, 6.5%; dyspnea, 4.2%
	77		RT	100	16.9	6.8	Grade ≥ 3 toxicity: radiation pneumonitis, 5.3%; dyspnea, 5.1%; infection, 4.1%)
Driessen et al. [19]	Patients	ΙΙΙ	CCRT	72	18	NA	NA
	≥ 70		SCRT	52	12		
			RT	34	11		
			NCT	58	5		
Miller et al. [20]	75.8	ΙΙΙ	CRT	18,206	18.1	NA	NA
	79.4		RT	5023	12.2		
Kim et al. [21]	73.0	ΙΙΙ	CCRT	54	21.1	73	Grade ≥ 2 esophagitis, 44.4%; Grade 3–4 radiation pneumonitis, 18.5%
	75.2		RT	28	18.1	75	Grade ≥ 2 esophagitis, 17.9%; Grade 3–4 radiation pneumonitis, 14.3%
Corre et al. [17]	83.9	I–IV	EGFR-TKI	114	20.9	11.9	Grade ≥ 3 diarrhea, 17.0%; cutaneous, 10.3%; others, 41.0%
Present study	82.0	ΙΙ–ΙΙΙ	CIRT	32	33.1	20.8	Grade 3 radiation pneumonitis, 3.1%
		(ΙΙΙ)		(15)	(22.2)	(15.1)	

*PFS* progression-free survival, *OS* overall survival, *CRT* chemoradiotherapy, *RT* radiotherapy, *CCRT* concurrent chemoradiotherapy, *SCRT*, sequential chemoradiotherapy, *NCT* no curative treatment, *NA* not applicable, *EGFR-TKI* an epidermal growth factor receptor tyrosine kinase inhibitor, *CIRT* carbon-ion radiotherapy

Our study had several limitations. First, the present study was a single-center retrospective analysis. Second, our results were derived from a small sample size of only 32 patients. Third, only octogenarians with Eastern Cooperative Oncology Group performance status of 0–2 were treated with CIRT alone, and thus there might have been patient bias. Finally, the total doses and fractionation varied (68–76 Gy (RBE) in 12–16 fractions) among the patients. Further large-scale multicenter prospective trials are warranted.

In conclusion, CIRT alone is a relatively safe and effective treatment modality for octogenarians with LA-NSCLC.
